# Clinical, dosimetric, and reporting considerations for Y-90 glass microspheres in hepatocellular carcinoma: updated 2022 recommendations from an international multidisciplinary working group

**DOI:** 10.1007/s00259-022-05956-w

**Published:** 2022-09-17

**Authors:** Riad Salem, Siddharth A. Padia, Marnix Lam, Carlo Chiesa, Paul Haste, Bruno Sangro, Beau Toskich, Kirk Fowers, Joseph M. Herman, S. Cheenu Kappadath, Thomas Leung, Daniel Y. Sze, Edward Kim, Etienne Garin

**Affiliations:** 1 Department of Radiology, Northwestern Feinberg School of Medicine, 676 N. St. Clair, Suite 800, Chicago, IL USA; 2 Department of Radiology, University of California-Los Angeles, Los Angeles, CA USA; 3 Department of Radiology and Nuclear Medicine, University Medical Center Utrecht, Utrecht, The Netherlands; 4 Department of Nuclear Medicine, Fondazione IRCCS Istituto Nazionale Tumori, Milan, Italy; 5 Department of Interventional Radiology, Indiana University School of Medicine, Indianapolis, IN USA; 6 Liver Unit, Clinica Universidad de Navarra and CIBEREHD, Pamplona, Spain; 7 Department of Radiation Oncology, Mayo Clinic, Jacksonville, FL USA; 8 Boston Scientific Corporation, Marlborough, MA USA; 9 Department of Radiation Medicine, Northwell Health, New Hyde Park, NY USA; 10 Department of Imaging Physics, University of Texas MD Anderson Cancer Center, Houston, TX USA; 11 Comprehensive Oncology Centre, Hong Kong Sanatorium and Hospital, Hong Kong, Hong Kong; 12 Department of Radiology, Stanford University School of Medicine, Palo Alto, CA USA; 13 Department of Interventional Radiology, Mount Sinai, New York City, NY USA; 14 INSERM, INRA, Centre de Lutte Contre Le Cancer Eugène Marquis, Institut NUMECAN (Nutrition Metabolisms and Cancer), Univ Rennes, 35000 Rennes, France

**Keywords:** Radioembolization, Yttrium-90, Dosimetry, Hepatocellular carcinoma

## Abstract

**Purpose:**

In light of recently published clinical reports and trials, the TheraSphere Global Dosimetry Steering Committee (DSC) reconvened to review new data and to update previously published clinical and dosimetric recommendations for the treatment of hepatocellular carcinoma (HCC).

**Methods:**

The TheraSphere Global DSC is comprised of health care providers across multiple disciplines involved in the treatment of HCC with yttrium-90 (Y-90) glass microsphere–based transarterial radioembolization (TARE). Literature published between January 2019 and September 2021 was reviewed, discussed, and adjudicated by the Delphi method. Recommendations included in this updated document incorporate both the results of the literature review and the expert opinion and experience of members of the committee.

**Results:**

Committee discussion and consensus led to the expansion of recommendations to apply to five common clinical scenarios in patients with HCC to support more individualized efficacious treatment with Y-90 glass microspheres. Existing clinical scenarios were updated to reflect recent developments in dosimetry approaches and broader treatment paradigms evolving for patients presenting with HCC.

**Conclusion:**

Updated consensus recommendations are provided to guide clinical and dosimetric approaches for the use of Y-90 glass microsphere TARE in HCC, accounting for disease presentation, tumor biology, and treatment intent.

## Introduction

Over the past decade, there has been an increasing use of transarterial radioembolization (TARE) using yttrium-90 (Y-90) glass microspheres in patients with hepatocellular carcinoma (HCC) across the Barcelona Clinic Liver Cancer (BCLC) classification spectrum. Since the previous publication of clinical and dosimetric considerations for TARE with Y-90 glass microspheres, additional published work has demonstrated the critical role of personalized dosimetry, optimizing dosing and patient selection in achieving improved clinical outcomes [1–3]. Additionally, several investigations demonstrated that increasing tumor-absorbed dose increases the likelihood of achieving complete pathologic necrosis, without compromising safety, while limiting toxicity by minimizing normal liver tissue exposure [4, 5]. These investigations have led to the inclusion of TARE in the updated BCLC staging system for treatment of single-HCC lesions ≤ 8 cm [6]. The decision to use single-compartment or multicompartment dosimetry in these studies underscores the importance of matching the appropriate TARE treatment approach with specific patient characteristics and treatment goals [1–3]. Given the number of new trials, recent publications, and their rapid impact on clinical practice, the TheraSphere Global DSC was reconvened to evaluate these new data and to update the recommendations [7].

## Methods

The committee, comprised of interventional radiologists, radiation oncologists, nuclear medicine physicians, clinical scientists, medical oncologists, and physicists, reconvened for four 2-h virtual meetings, with an additional offline review in preparation for the process of updating the recommendations. During the first meeting, the committee reviewed the prior recommendations, recently published literature, and discussed updates. To identify literature since the prior recommendations, a PubMed search using a combination of the following search terms was conducted: transarterial radioembolization, TARE, brachytherapy, internal radiation therapy, SIRT, Y90, yttrium-90, TheraSphere, hepatocellular carcinoma, and HCC. The committee also considered whether new data warranted changing the degree of recommendation and/or the strength of consensus from the previous recommendations (Tables 1 and 2). Briefly, per the Delphi method, consensus was defined during virtual meetings as outlined in Table 2; strong disagreements by members were recorded and highlighted within the recommendation as caveats, where applicable. During the second and third meetings, committee members reviewed the revised recommendations and discussed each change collectively. Between the meetings, the lead author (RS) revised the recommendations based upon committee discussion and comments. The fourth and final meeting was a comprehensive review of the recommendations and a review of the draft manuscript. Steering committee members then had the opportunity to review and refine the manuscript independently, and final comments were incorporated into the manuscript by the lead author. All authors formally endorsed the manuscript and its recommendations prior to submission.

**Table 1:** Degree of recommendation

Degree	Meaning
A	Strongly recommended (good evidence that the measure is effective, and benefits outweigh the harms)
B	Recommended (at least moderate evidence that the measure is effective and that benefits exceed harms)
C	No recommendation for or against (at least moderate evidence that the measure is effective, but benefits are similar to harms and a general recommendation cannot be justified)
D	Recommended against (at least moderate evidence that the measure is ineffective or that harms exceed the benefits)
E	Insufficient, low quality, or contradictory evidence (the balance between benefit and harms cannot be determined)

**Table 2:** Strength of consensus

Strength of consensus	Definition
Strong	≥ 80% consensus
Moderate	50–79% consensus
Weak	≤ 49% consensus

The reviewed literature included all published studies of TARE with glass microspheres for HCC since January 1, 2019; additional studies outside of this timeframe were reviewed if suggested by steering committee members. Publications addressing technical challenges rather than clinically oriented approaches were not included in the review. The recommendations included in this updated document incorporate both the critical literature review and the expert opinion and experience of members of the committee. All recommendations made in Tables 3, 4, 5, 6, and 7 are subject to regulatory and clinical standards within each country.

**Table 3:** Scenario 1: radiation segmentectomy recommendations using Y-90 glass microspheres

Treatment intent	Definitive therapy if non-transplant candidate (ex: solitary T1, solitary/multifocal UNOS T2–T3). Tumor control with potential for additional curative treatment in appropriately selected patients (ex: solitary/multifocal UNOS T1–T2–T3) for bridging/downstaging to transplantation
Patient selection	1. Child–Pugh A and select B7, tumor stage UNOS T1–T3 (may consider Child–Pugh B7-C [rare scenario] if bridging or downstaging to transplant and segmental infusion possible) [5, 15, 29, 54–58] 2. Treatment may be performed in patients with prior liver therapy (i.e., surgical resection, ablation, external beam radiation therapy, or stereotactic body radiation therapy) [5, 15, 56, 59]. In patients with prior chemoembolization, angiographic assessment of vascular supply and patency during mapping angiography will determine TARE eligibility. While patients can receive Y-90 glass microsphere TARE after external beam radiation therapy or stereotactic body radiation therapy, more data is needed to determine efficacy and safety. Early data suggests it is safe in patients with preserved liver function 3. Tumors abutting the colon, gallbladder, and stomach can be safely treated; radiation toxicity in this specific setting of adjacent structures has been reported but is extremely rare [60] 4. Multiple radiation segmentectomy infusions in two separate Couinaud segments may be performed for multifocal disease during the same session, including two segments that would define a bilobar disease in patients with normal underlying liver or well-compensated cirrhosis (ex: 1 lesion in segment 6, 1 lesion in segment 2) [15, 16]. Historically, radiation segmentectomy was defined as ≤ 2 segments; however, current definitions include infusion of Y-90 glass microspheres to much smaller segments of liver, referred to as angiosomes, with the intent of delivering ablative dose to tissue. Recent investigations have reported Y-90 glass microsphere TARE infusion in up to 25% in ALBI-1 with excellent tolerability and noted additional liver toxicities above 14% in ALBI-2 and Child–Pugh B patients [17] 5. In patients with previous hepatectomy, the choice to use radiation segmentectomy should be approached with caution considering remaining FLR and potential toxicity. Pretreatment considerations would include the magnitude of post-hepatectomy hypertrophy, time from resection to recurrence, and the total volume of liver parenchyma. Therefore, the use of radiation segmentectomy in this setting requires further investigation
Treatment planning	
Diagnostic studies and target volume definition	Diagnostic imaging should ideally be multiphase contrast-enhanced magnetic resonance (MR) [61]; contrast-enhanced computed tomography (CT) can also be used. Both imaging modalities are considered acceptable 1. Determine angiosome volume by cone-beam CT; this is the gold standard for perfused volume determination and preferred method when available [3, 18, 19] 2. If there is associated segmental portal venous invasion, treat the territory that encompasses the MVI/PVT confirmed by cone-beam CT [15] 3. If there is a suspicion of microsatellite lesions, treat a wider territory (i.e., the larger the lesion, the wider the safety margin necessary) confirmed by cone-beam CT; an angiographic/cone-beam CT margin of ≥ 1 cm is recommended [62]
Mapping and ^99m^Tc-MAA	1. The need for prophylactic embolization is very low (unless distal branch from infusion site leads to the gastrointestinal tract) (e.g., left hepatic artery injection with accessory left gastric artery arising distally, left hepatic artery injection with esophageal branch arising distally) [63] 2. Perform lobar technetium-99 m macroaggregated albumin (^99m^Tc-MAA) and segmental Y-90 infusion to limit the number of catheterizations of the small segmental branch perfusing tumor [16] 3. Elevated lung shunt fraction limiting the intended dose is rarely an issue because of minimal tumor load (low shunting) and limited prescribed activities (small, perfused volumes) [16]. In the case of small tumors (i.e., those less than 5 cm) and in the absence of MVI/PVT, the risk of high lung shunt is low. In such cases, it may be possible to eliminate the ^99m^Tc-MAA mapping step from the treatment planning process [16, 64]; however, more studies evaluating this concept are needed. In such cases, dosimetry is still required for dose determination. No formal recommendation on eliminating the ^99m^Tc-MAA can be made at this time
Dose calculation and dosimetry considerations	1. Single-compartment model dosimetry is adequate and preferred [3] 2. Target-absorbed dose to the perfused treatment volume of at least 400 Gy to the angiosome with no established upper limit. A median of 400 Gy resulted in 100% of patients achieving complete pathologic necrosis in tumor explants [3, 4]. Similar results using > 500 Gy to the perfused volume were reported [29]. Prospective validation demonstrates an adverse event profile that is minimal using this approach [20] 3. Recent publications have demonstrated that higher doses to the segment ≥ 400 Gy yield better pathologic and clinical outcomes [3, 16, 42]. An upper threshold dose limit may exist, but it is currently unknown based on the available literature. In case of a small, treated volume, the dose is oftentimes determined by the lowest available vial (i.e., 3 GBq at calibration) 4. Recommend week 1 (Wednesday/Thursday/Friday) or early week 2 dosing (Monday/Tuesday) to replicate published outcomes [3, 11]. With glass microspheres, there is preliminary data to suggest that late first-week and early second-week microsphere-specific activity (estimated ≥ 297 Bq) may be associated with increased pathologic necrosis in small tumors treated with radiation segmentectomy [1, 42]
Treatment delivery	1. Ensure no contrast refluxes into an adjacent angiosome prior to treatment 2. The entire tumor (and microsatellites) should lie within the perfused angiosome 3. Prime the TheraSphere® injection system slowly a). There is a low margin of error in radiation segmentectomy given the small territory b). Prime the system slowly to minimize the risk of bubble formation 4. Consider a 2.1/2.4 French (or smaller) microcatheter in a segmental branch. Exercise caution if using smaller than 2 French due to a risk of incomplete administration [12, 65] 5. Same-day planning ^99m^Tc-MAA and treatment approaches may be considered (i.e., low activity administration needed for high absorbed dose, with a very low chance of high lung-absorbed dose) [16, 64]
Outcome assessment/follow-up	1. Ideally, use the same imaging modality that was used for initial assessment of disease burden (contrast-enhanced CT or multiphase contrast-enhanced MR) 2. If complete mRECIST response at 3–6 months is not achieved, consider retreatment [3, 15, 66, 67]
Strength of recommendation	A
Degree of consensus	Strong

**Table 4:** Scenario 2: radiation lobectomy recommendations using Y-90 glass microspheres

Treatment intent	To increase the number of patients who can undergo curative surgical resection given limited organ availability for liver transplantation (ex: UNOS T2-T3, unilobar T4a) [21–24]
Patient selection	1. Radiation lobectomy applies to unresectable Child–Pugh A patients in the following scenarios: a) Inadequate FLR and/or b) Test of time is desired for tumor biology and response prior to surgery and/or c) Need for the treated tumor to be retracted away from hepatic vein and/or IVC d) Potential delay of surgery or definitive treatment instead of surgery 2. Borderline resectable patients are considered, and therefore should not have comorbidities that would preclude surgery
Treatment planning	
Diagnostic studies and target volume definition	1. Contrast-enhanced cone-beam CT in the angiography suite should be performed to assess/ensure tumor coverage within the treated lobe
Mapping and ^99^mTc-MAA	a) Perform lobar ^99^mTc-MAA and lobar Y-90 infusion. Catheter placements should be to facilitate similar distribution pattern b) Elevated lung dose may be an issue if the lung shunt fraction is high in the context of large perfused volume
Dose calculation and dosimetry considerations	1. Using a multicompartment model with ^99m^Tc-MAA, a recent randomized study demonstrated that tumor response in patients with ≥ 30% hepatic reserve is optimized and overall survival extended when the minimum planned tumor-absorbed dose is ≥ 205 Gy (with a mean of 331 Gy) and normal tissue–absorbed dose (NTAD) is ≤ 120 Gy attained by treating on week 1 (Wednesday) [1]. A minimum threshold absorbed dose of normal injected liver > 88 Gy with week 1 (Wednesday) dosing in Child–Pugh A patients ensures a minimum 10% hypertrophy [25]. As an alternative planning criterion, a retrospective study of normal tissue complication probability determined the maximum tolerable dose for Child A patients at 50 Gy or 90 Gy whole non-tumoral liver (including perfused and non-perfused normal liver) with a bilirubin level ≥ 1.1 mg/dL or < 1.1 mg/dL, respectively, using 4-day decay, to minimize hepatic dysfunction [41] 2. If using a single-compartment model, a 140–150 Gy lobar absorbed dose limit is recommended given implied Child–Pugh A status for radiation lobectomy patients [21, 25]. A recent randomized study demonstrated that for well-selected patients (Child–Pugh A and hepatic reserve > 30%), targeting a lobar absorbed dose > 150 Gy (with a mean of 178 Gy) with a whole liver dose < 150 Gy, by treating on week 1 (Wednesday), for well-selected patients (Child–Pugh A and hepatic reserve > 30%) was safe and can be used [1]. Retreatment should be considered if minimal hypertrophy is noted at months 1–3 3. Existing literature supports treatment on week 1 (Wednesday) to week 2 (Tuesday). No optimal day has been identified [21, 22, 68] 4. Repeated treatment of the same volume has been performed and is safe when carefully considering dosimetry and liver function
Treatment delivery	1.Radiation lobectomy is most commonly encountered with right lobe HCC. Treat the right lobe tumor and induce left lobe hypertrophy in anticipation of surgery [21, 22, 24, 69, 70] 2.Treatment should be administered in a lobar manner (i.e., such that the entire lobe is treated). If segmental treatment might otherwise be technically feasible but the goal is for contralateral lobar hypertrophy to bridge to resection, one can consider “modified” radiation lobectomy, where a single-session segmental tumor infusion (single-compartment dose to segment ≥ 400 Gy; radiation segmentectomy, see previous section) is followed by lobar infusion, with the second vial delivering single-compartment 100 Gy to the lobe for hypertrophy [3, 21, 71] a) Modified radiation lobectomy is favored over single lobar infusion when technically feasible b) In the setting of a), if patient does not undergo surgery, tumor control has been maximized by performing curative high absorbed dose segmentectomy treatment
Outcome assessment/follow-up	1. Imaging with dynamic assessment of FLR is recommended at 1 month, 3 months, 6 months, and 9 months after treatment. Tumor volume should be subtracted from total right lobe volume when calculating FLR 2. Allow at least 3–6 months for hypertrophy; a longer wait time is acceptable as long as the tumor is well controlled [25, 27, 69] 3. Portal vein embolization after lack of hypertrophy from Y-90 radioembolization is currently investigational [22, 24]. Radioembolization after portal vein embolization is also investigational [22] 4. Pre- and post-TARE hepatobiliary scintigraphy or Eovist® (USA) or Primovist® (EU) (gadolinium-EOB-DTPA) using MRI to further determine if adequate FLR was attained, if additional treatment is required, or if the patient is ultimately suitable for subsequent surgical resection is investigational [27–29] 5. The decision to proceed with resection post TARE is jointly decided upon with surgeons. In some cases, resection may be deemed unnecessary given complete tumor response and radiation lobectomy becomes definitive treatment [21, 24]
Strength of recommendation	B
Degree of consensus	Strong

**Table 5:** Scenario 3: multifocal unilobar HCC without macrovascular invasion recommendations using Y-90 glass microspheres

Treatment intent	Palliation and delaying disease progression ahead of initiation of systemic therapy or downstaging to resection. The goal should be to provide optimal tumor-absorbed dose and keep NTAD below a safe ceiling for the following reasons: a) Many patients are treated with palliative intent due to a multifocal disease within a single lobe b) Liver function should be preserved in order that subsequent treatment is potentially possible (e.g., surgery after downstaging, repeat radioembolization, chemoembolization, local ablative therapies, systemic therapy) [21, 22, 72]
Patient selection	1. Patients should have Child–Pugh A or B7 cirrhosis. The committee recommends that multidisciplinary discussions and individualized patient characteristics be considered prior to considering treatment with Y-90 glass microspheres, especially in patients more severe hepatic dysfunction [1, 23, 30–32, 45, 46]
Treatment planning	
Mapping and ^99m^Tc-MAA	Injection of ^99m^Tc-MAA in the lobar hepatic artery in order to perfuse the entire lobe
Dose calculation and dosimetry considerations	1. If possible, multicompartment dosimetry model is preferred over a single-compartment model to maximize tumor-absorbed dose and evaluate normal parenchyma absorbed dose [1, 41] 2. In a multicompartment model, prediction of the normal liver absorbed dose is typically more accurate than the tumor-absorbed dose, especially for small tumors. A recent randomized study demonstrated that tumor response in patients with ≥ 30% hepatic reserve is optimized and overall survival extended when the minimum tumor-absorbed dose is ≥ 205 Gy, with > 250 Gy where possible (with a mean of 331 Gy), and NTAD is ≤ 120 Gy attained by treating on week 1 (Wednesday) [1]. Although there are several investigations looking into the upper limit of dose to normal parenchyma averaged over the whole liver (examples, 50 Gy or 90 Gy whole liver with a bilirubin level ≥ 1.1 mg/dL or < 1.1 mg/dL, respectively, using 4-day decay), this continues to be investigational [41] 3. Optimal tumor-absorbed dose (i.e., dose associated with response) is ≥ 205 Gy, with > 250 Gy where possible (with a mean of 331 Gy) [1, 32, 39, 55, 73]. This is only feasible if the multicompartment model can be applied. Recent publications demonstrated that tumor response and median overall survival improved with increasing tumor-absorbed dose [1, 2]
Treatment delivery	1. Single-compartment dosimetry supports 120–150 Gy to the perfused lobe [1, 13] 2. Multicompartment dosimetry supports a minimum tumor-absorbed dose of ≥ 205 Gy, with > 250 Gy where possible (with a mean of 331 Gy) treating on week 1 (Wednesday) [1]. Treatment between week 1 (Wednesday) and week 2 (Tuesday) is acceptable 3. The decision on perfused volume or tumor and NTAD should be based on treatment intent relative to clinical status, liver function, tumor load, targeting, vascularity, and previous treatments [39]
Outcome assessment/follow-up	1. Multiphase CT or MR should be performed every 3 months following treatment with consideration for FLR, hypertrophy, candidacy for surgical resection, and/or systemic therapy. In the palliative intent setting, caution is warranted with an overly aggressive approach to retreatment in patients with stable disease or partial response. Retreatment in the form of radioembolization, chemoembolization, or systemic therapy should typically be considered only in the setting of progressive disease. Empirically initiating systemic therapy following partial or complete response, or stable disease, remains investigational and should be individualized
Strength of recommendation	B
Degree of consensus	Strong

**Table 6:** Scenario 4: multifocal bilobar HCC without macrovascular invasion recommendations using Y-90 glass microspheres

Treatment intent	Palliation and delaying disease progression. The goal should be to provide sufficient tumor-absorbed dose and keep NTAD dose below a safe ceiling for the following reasons: a) Most patients are treated with palliative intent due to late-stage disease with diffuse multifocal lesions with or without large tumor load in both lobes requiring higher exposure to normal tissue to effectively treat [35] b) Liver function should be preserved to permit subsequent treatment using repeat radioembolization, chemoembolization, or systemic therapy [24, 35, 36]
Patient selection	1. Bilobar HCC patients for Y-90 should have Child–Pugh A cirrhosis and appropriate performance status. At least 30% hepatic reserve is ideal [45, 46]
Treatment planning	
Mapping and ^99m^Tc-MAA	1. Multiple variations of ^99m^Tc-MAA administration exist. Options include: a) Injection of ^99m^Tc-MAA in the proper hepatic artery in order to perfuse the entire liver b) Injection in the lobe with higher tumor burden (yields most conservative estimate) c) Injection in both lobes with a split vial of ^99m^Tc-MAA into RHA and LHA, respectively d) Sequential lobar infusion of ^99m^Tc-MAA requiring 2 separate mapping angiogram procedures on separate days (most accurate for multicompartment dosimetry)
Dose calculation and dosimetry considerations	1. A multicompartment dosimetry model is preferred over single compartment to evaluate normal parenchyma–absorbed dose relative to treatment intent [1, 35, 41] 2. In a multicompartment model, prediction of the normal liver absorbed dose is typically more accurate than the tumor-absorbed dose, especially for small tumors [41, 43]. Targeting from 40 to 70 Gy absorbed dose to the entire normal liver tissue may be performed safely in a Child–Pugh A patient [35, 74–77]. Additional data is needed to identify the appropriate post-calibration day of treatment 3. Contemporary techniques use multicompartment dosimetry in this population to achieve optimal results [35, 47]. Optimal tumor-absorbed dose (i.e., dose associated with response) is ≥ 205 Gy, with > 250 Gy where possible (with a mean of 331 Gy) [1, 32, 39, 55, 73]. This is only feasible if the multicompartment model can be applied 4. Single-compartment dosimetry supports 120 Gy (range 80–150 Gy) to the perfused tissue [13]. The decision on absorbed dose should be based on clinical status, liver function, tumor load, targeting, vascularity, and previous treatments
Treatment delivery	1. To treat bilobar disease, the treating physician has the discretion to choose single-session bilobar or staged sequential lobar treatment [35]. In general, staged sequential lobar treatment is preferred and the lobe with more extensive disease should be treated first. Second treatment, if stage approach is adopted, is recommended at 4–8 weeks once liver function tests are assessed [31, 41, 47, 78]. For highly aggressive bilobar disease in a patient with Child–Pugh A cirrhosis and with good tumor targeting on ^99m^Tc-MAA (i.e., high tumor-absorbed dose; low normal liver absorbed dose), single-session bilobar treatment (2 unilobar injections) based on multicompartment dosimetry can be considered [35, 47]. Multidisciplinary discussions are recommended to include the use of systemic therapy in aggressive biology disease.
Outcome assessment/follow-up	1. Multiphase CT or MR should be performed every 3 months following treatment. Given the palliative intent in this setting, caution is warranted with an overly aggressive approach to retreatment in patients with stable disease or partial response. Retreatment in the form of radioembolization, chemoembolization, or systemic therapy should typically be considered only in the setting of progressive disease. Empirically initiating systemic therapy following partial or complete response, or stable disease, remains investigational and should be individualized
Strength of recommendation	B
Degree of consensus	Strong

**Table 7:** Scenario 5: HCC with macrovascular invasion recommendations using Y-90 glass microspheres

Treatment intent	Palliation and enabling disease control, combining and/or bridging to systemic treatment. Surgical conversion or downstaging may be possible [1, 34, 35]
Patient selection	1. Child–Pugh A patients with good tumor and MVI/PVT targeting and low NTAD can be considered when locoregional therapy is selected prior to the initiation of systemic therapy [1, 34–36, 45, 46]. Those with unilobar MVI/PVT should be considered for TARE, with early consideration for systemic therapy. Patients with bilobar MVI/PVT should be considered for upfront systemic therapy, especially if associated with CP B disease; these patients are unlikely to benefit from initial treatment with TARE 2. Treatment can be considered for segmental, lobar, or branch MVI/PVT, with follow-up imaging dictating when to consider adding systemic therapy. For main MVI/PVT with good targeting, ≥ 30% hepatic reserve, and unilobar treatment, some patients may benefit from TARE; however, early (1 month) post-Y-90 combination with systemic agents may be an option for this population [1, 34, 35, 37–39] 3. Larger tumors (e.g., > 10 cm) with MVI/PVT have been effectively treated with glass microsphere TARE using multicompartment dosimetry [1, 37, 39]
Treatment planning	
Diagnostic studies and target volume definition	1. Treatment should be performed in a location that is proximal enough to perfuse all feeding vessels both into the tumor and to the tumor thrombus. The use of contrast-enhanced cone-beam CT during angiographic mapping can aid in detection of MVI/PVT perfusion
Mapping and ^99m^Tc-MAA	1. ^99m^Tc-MAA MVI/PVT targeting evaluation should be performed [1, 39, 79]
Dose calculation and dosimetry considerations	1. Multicompartment dosimetry is preferred to maximize sparing of normal parenchyma [1, 35, 40]. This approach may be challenging in the setting of infiltrative disease, where tumor and normal parenchyma cannot be differentiated 2. For the multicompartment model, a recent randomized study demonstrated that tumor response in patients with ≥ 30% hepatic reserve is optimized and overall survival extended when the minimum tumor-absorbed dose is ≥ 205 Gy, with > 250 Gy where possible (with a mean of 331 Gy), and NTAD is ≤ 120 Gy, attained by treating on week 1 (Wednesday) [1]. The ideal candidate has good MVI/PVT ^99m^Tc-MAA targeting (treatment intensification), as a suboptimal response is likely if there is no ^99m^Tc-MAA MVI/PVT targeting or tumor-absorbed dose is < 205 Gy [39]. In such cases, advanced angiographic techniques may be attempted, e.g., boost dosing, if specific vessels can be identified. The use of systemic therapy in patients without significant uptake on MAA should also be strongly considered [1, 40]. Multicompartment dosimetry with good MVI/PVT and tumor targeting may be considered to downstage patients to surgery. Preservation of FLR function is a key consideration [1, 39]
Treatment delivery	1. An aggressive dosing approach (similar to radiation lobectomy) can be used for unilobar disease and Child–Pugh A liver function if lung shunt fraction permits 2. A more conservative approach, including treatment planning using multicompartment dosimetry, or consideration of systemic therapy, should be used for bilobar disease (similar to patients with multifocal bilobar HCC), especially when portal perfusion of a large portion of the functional liver is compromised by tumor invasion [35]
Outcome assessment/follow-up	Multiphase CT/MR should be performed every 3 months following treatment. Systemic therapy or enrollment into clinical trials should be considered for patients who not only demonstrate progression but should also be considered in the setting of stable disease in order to prolong time to progression and capitalize on the combination effect of locoregional and systemic therapies. Given the palliative intent in this setting, caution is warranted with an overly aggressive approach to retreatment in patients with stable disease or partial response
Strength of recommendation	A
Degree of consensus	Moderate

In light of the changing paradigms and treatment goals associated with Y-90 glass microsphere TARE, the committee agreed to expand the clinical scenarios from four to five, separating multifocal unilobar and bilobar disease recommendations, and to revise the definition of each. The scenarios subsequently addressed in the updated recommendations are as follows:

Curative intent:

- *Radiation segmentectomy*: Localized disease (one or multiple tumors located in ≤ 2 segments), with contemporary and modern treatment approaches delivering superselectively to subsegments of liver, referred to as angiosomes (i.e., hepatic territory perfused by a specific branch of the hepatic artery), with the intent of delivering an ablative dose to tumor and normal tissue. Radiation segmentectomy no longer narrowly defined as ≤ 2 segments but rather inclusive of smaller hepatic segmentectomy
- *Radiation lobectomy*: Unilobar disease, with the ultimate goal of disease control and contralateral lobar hypertrophy in the context of small future liver remnant (FLR), as a bridge to surgery (resection)

Palliative intent:

- Multifocal unilobar disease without macrovascular invasion or portal vein thrombosis (MVI/PVT), with the goal of palliation and delay in progression; in select patients, intent may be conversion to curative options
- Multifocal bilobar disease without MVI/PVT, with the goal of palliating and delaying progression, usually in combination or in sequence with systemic treatment
- HCC with MVI/PVT, with the goal of palliating and delaying progression; in select patients, intent may be conversion to curative options

Key definitions used throughout this document were defined in the original publication and are reprinted below for ease of reference [7]:

- *Mean absorbed dose*: Quantity is expressed in gray (Gy) in order to describe the average energy (J) deposited within a volume of interest (VOI) within a specific given mass (kg). The mean absorbed dose is referred to as “Dose” and is distinctly different than “Activity” or “Dosage” (GBq) [8, 9].
- *MIRD schema*: The Medical Internal Radiation Dose (MIRD) schema is applicable to both the single-compartment and multicompartment dosimetry models. The mean absorbed dose (*D*) in any specific VOI (i.e., perfused volume, lobe, tumor or normal tissue) with mass of any VOI, denoted as *M*, with the assumption that *D* is distributed uniformly in each volume with permanent microsphere implantation and no biological clearance [10, 11]. Using this schema, *D* in a VOI is computed as:
  where *A* is the net activity of ^90^Y implanted in the VOI, and *F* is the lung shunt fraction. As an example, if 2.2 GBq of glass microspheres was infused with a residual of 1% and a lung shunt of 5%, the net implanted activity in the liver tissue would be 2.2 × (0.99) × (0.95) = 2.07 GBq, and 2.07 GBq would represent the final activity within a MIRD formula for determining final tissue dose.
  $${D}_{\left(\mathrm{Gy}\right)}=\frac{{A}_{(\mathrm{GBq})}\times ({50}_{(\mathrm{Gy}/\mathrm{kg}/\mathrm{GBq})}\left(1-F\right))}{{M}_{(\mathrm{kg})}}$$
- *Single-compartment model*: A MIRD dosimetry model that assumes the ^90^Y microspheres (and therefore absorbed dose) are distributed uniformly within the VOI. In this model, only a uniform averaged *D* value over the VOI is calculated, without consideration of Y-90 activity distribution within the tumor and normal parenchyma. In reality, hypervascular tumors will absorb more microspheres and receive a higher dose, while the normal hepatic tissue will absorb fewer spheres and receive a lower dose [12–14].
- *Multicompartment model*: A MIRD-based dosimetry approach where *D* is determined in more than one VOI, such as the tumor VOI and the normal parenchyma VOI. The lung also represents another compartment to which *D* can be estimated (based on a single-compartment model). Partition modeling refers to the multicompartment dosimetry approach reporting the tumoral and non-tumoral doses separately with a single averaged tumor to averaged non-tumoral uptake ratio (T:N ratio) [10].

## Results

Relevant publications from the literature review, including additional suggestions by the committee, resulted in the inclusion of 31 new publications [1–6, 16, 17, 19, 20, 24–26, 28–31, 35, 36, 41, 47, 48, 50–58] and formed the basis for updates to the recommendations.

### Clinical scenarios

#### Scenario 1: radiation segmentectomy for HCC

Radiation segmentectomy has been previously defined as the administration of Y-90 to ≤ 2 Couinaud segments with curative intent. In practical terms, this translates to subselective or superselective radioembolization. In most scenarios, a superselective, subsegmental infusion covering significantly ≤ 1 is achieved [15, 16]. For radiation segmentectomy, the percentage total liver volume that is treated should be considered. As long as a minimal amount of tissue is exposed, radiation segmentectomy may be considered in the setting of prior surgical resection (particularly if robust post-surgical hypertrophy has been observed). In the presence of stable but reduced hepatic function, radiation segmentectomy should be undertaken using caution and consideration of alternate options [3, 17–19]. Details are included in Table 3.

The objective is ablative dosing for both tumoral and non-tumoral compartments as both are expendable in this scenario [3]. The strength of these recommendations was an A in the previous version and remains an A with a strong degree of consensus from the committee; this recommendation was further reinforced by the findings of the prospective RASER study [20]. Partition dosimetry was not recommended by the panel in this scenario.

#### Scenario 2: radiation lobectomy for HCC

In unilobar HCC patients, resection is often not possible due to small FLR. For these patients, radiation lobectomy may be an option, as delivery of a high dose of radiation to one lobe may trigger the hypertrophy-atrophy complex, inducing hypertrophy in the contralateral lobe and thereby increasing the functional liver volume while controlling tumor growth in the treated lobe (Table 4) [21–24]. The increased FLR may allow for subsequent curative resection.

Depending on patient biology and mapping results, either single-compartment or multicompartment dosimetry approaches may be used [1, 21, 25, 26]. Hepatobiliary scintigraphy (HBS), including ^99m^Tc-iminodiacetic acid (HIDA) or ^99m^Tc-mebrofenin, and the use of Eovist/Primovist contrast media in magnetic resonance imaging are emerging investigational techniques that are being implemented for regional functional assessment of the FLR before and after Y-90 treatment. Additional data are needed to support a strong recommendation of their use as a standard of care in radiation lobectomy and resection planning [27–29]. The strength of these recommendations was previously a B with strong consensus from the committee; despite these additional studies and data, the committee deemed the data to be insufficient to raise the grade to A and maintained the grade of B with strong consensus.

#### Scenario 3: multifocal unilobar HCC without macrovascular invasion (MVI/PVT)

For patients with multifocal unilobar disease without MVI/PVT, radioembolization can be used for palliation and prevention of disease progression (Table 5). In this population, the goal should be to maximize tumor dose while preserving liver function. The committee recommends that radioembolization be used for Child–Pugh A patients and that a multidisciplinary tumor board review be conducted, especially for Child–Pugh B patients who have more severe hepatic dysfunction [1, 23, 30–32]. In select cases, this group of patients may be considered for resection if they exhibit responses and hypertrophy features of radiation lobectomy.

Previously, multifocal unilobar and bilobar diseases were grouped together and granted a B grade with a strong committee consensus; this newly created unilobar disease section has been given a grade of B with strong committee consensus.

#### Scenario 4: multifocal bilobar HCC without MVI/PVT

In multifocal bilobar HCC without MVI/PVT, the primary goals of treatment are frequently palliation, delaying disease progression, and sequencing with other liver-directed therapies and/or systemic treatment (Table 6). As with other clinical scenarios, the goal should be to maximize the dose of radiation delivered to the tumor while minimizing the dose to remaining normal liver parenchyma. Previously, multifocal unilobar and bilobar diseases were grouped together and given a grade of B with a strong committee consensus; this newly created bilobar disease section has been given a grade of B with strong committee consensus.

#### Scenario 5: HCC with MVI/PVT

Patients with portal vein thrombosis (MVI/PVT), indicative of advanced HCC, generally have a poor prognosis. Such patients are not usually considered transplantation or resection candidates and may not achieve optimal outcomes with chemoembolization [1, 33–35]. With careful patient selection and dosimetric planning, radioembolization may achieve a long-term durable response without compromise of hepatic function in this population (Table 7) [1, 34–39]. The committee recommended a shift in defining which patients with MVI/PVT should be selected for treatment with Y-90 glass microsphere TARE, narrowing from previous broader recommendations to those who are Child–Pugh A5 or A6 (except in the case of segmental MVI/PVT where radiation segmentectomy may be considered [1, 34–36]. Multicompartment dosimetry is preferred in these patients to ensure that the maximum tumor-absorbed dose (TAD) is achieved while minimizing the dose to the normal tissue–absorbed dose (NTAD) and allowing the assessment of MVI/PVT targeting evaluation during pretreatment planning [1, 35, 40]. As with the approaches discussed earlier, an adequately high specific activity (the amount of radioactivity per microsphere at the time of administration) is important to achieve a desired TAD in the MVI/PVT with potentially limited microsphere deposition [1]. Given data from this and other recent studies, the committee chose to increase the degree of recommendation from B to A, with a moderate degree of committee consensus.

## Discussion

Results from over 30 manuscripts and abstracts published since 2019 prompted an update to treatment recommendations for Y-90 glass microsphere–based TARE in HCC patients; these included the DOSISPHERE-01, LEGACY, and TARGET studies [1–3]. While previous studies highlighted the improved overall survival in patients achieving complete response upon imaging, data from the recent DOSISPHERE-01 and TARGET studies further established associations between TAD, tumor response, and overall survival [1, 2]. For patients with limited disease, ablative Y-90 TARE remains the most effective and well-tolerated treatment option in eligible patients. Important new updates to the recommendations based on recent publications include more specific dosimetric recommendations for radiation segmentectomy and lobectomy, separating multifocal unilobar and bilobar diseases into different sets of recommendations and providing context in which Y-90 glass microsphere TARE should be used for patients with portal vein thrombosis. Additional multicompartment dosimetry updates included proposed new thresholds for tumor and NTAD and incorporated the impact of underlying liver function [3, 4, 41–43]. Multiple publications focused on the utility of ^99m^Tc-MAA imaging to estimate Y-90 glass microsphere distribution confirm the distribution of treatment and whether to select tumor or NTAD as the primary driver in choosing the appropriate Y-90 activity using multicompartment dosimetry [1, 41, 43].

### Ablative dosing approaches in radiation segmentectomy

Recent publications, including LEGACY and its companion publications, have reported on higher selective treatment-absorbed doses for radiation segmentectomy [3, 4, 42]. Higher absorbed dose in selective ablative Y-90 glass microsphere TARE led to increased rates of complete pathologic necrosis, e.g., ≥ 400 Gy; complete and extensive pathologic necrosis have been shown to be associated with reduced recurrence in patients bridging to transplant [5, 42]. However, a maximum perfused volume-absorbed dose has not yet been identified. Recent publications also refined guidance for albumin-bilirubin (ALBI)-1/Child–Pugh A and ALBI-2/Child–Pugh B patients from up to 2 Couinaud segments to specific volume recommendations [17]. The use of cone-beam CT or angio-CT with selective intra-arterial contrast enhancement provides the best preprocedural volume definitions for accurate dosimetry calculations, provides the most accurate arterial flow assessing for non-target flow and coverage of microsatellites, and ensures dose target accuracy [19].

### Improving conversion to resection

Recent studies and published recommendations have demonstrated the utility of personalized dosimetry in converting unresectable patients to candidates for resection [1, 3, 4, 21, 23, 25, 44]. Some recent publications showed that such an approach not only increased overall tumor response, but approximately doubled response rates when the TAD exceeded 300 Gy [1, 3]. In one of these investigations, multicompartment dosimetry used in multifocal unilobar HCC with or without MVI/PVT offered superior conversion of unresectable HCC compared to standard lobar single-compartment dosimetry (36% versus 4%, respectively) [1]. Single-compartment dosimetry using radiation lobectomy or modified lobectomy imparts local tumor control and contralateral lobe hypertrophy as a bridging strategy prior to resection. Collectively, these data demonstrated that treatment efficacy outcomes exhibit a continuum of improvement as TAD is escalated [3, 4, 21, 23]. Select studies provide guidance on the NTAD maximum and support evaluation of liver function, i.e., baseline bilirubin, prior to selecting NTAD targets [41]. Treatment outcome data regarding the use of NTAD to guide Y-90 glass microsphere activity selection are limited; further clinical data is needed to strengthen recommendations.

### Differing treatment approaches for unilobar and bilobar HCC

Based on approvals of new systemic treatment options, the committee decided to divide unilobar and bilobar diseases without MVI/PVT into two separate clinical scenarios [30]. Systemic therapy is the current standard of care for advanced disease; however, it is important to consider TARE early in treatment planning, as it plays an important role in providing a cytotoxic effect, while ensuring tumor control, preserving liver function, and keeping future treatment options available [45, 46]. In these patients, multicompartment dosimetry is preferred to adequately assess TAD and NTAD relative to the extent of disease and liver function. Select centers have published Y-90 glass microsphere TARE experience in patients undergoing bilobar treatment, demonstrating that a multicompartment dosimetry approach is appropriate and beneficial in bilobar HCC [35, 43, 47].

The NTAD values proposed for unilobar treatment, 50–90 Gy based on baseline bilirubin levels, are higher than those recommended for bilobar patients, 40–70 Gy. However, additional clinical data are needed to better define the appropriate range for bilobar patients [41, 43]. In the case that multicompartment dosimetry is not feasible and single-compartment dosimetry is used, a lower target (i.e., 120 Gy to the perfused volume) is recommended for bilobar HCC patients; planning for such treatment should include careful evaluation of clinical status and liver function when evaluating possible treatment options. A more conservative approach to TAD and NTAD is recommended in the palliative setting as compared to in patients where treatment intent includes downstaging or conversion to resection.

### Treatment goals for HCC with MVI/PVT

In HCC patients with MVI/PVT, treatment goals (i.e., potential downstaging or conversion to resection) and careful patient selection should drive the decision as to whether TARE should be used as monotherapy or in conjunction with systemic treatment. In both DOSISPHERE-01 and TARGET, Y-90 glass microsphere TARE was evaluated in patients with MVI/PVT as monotherapy [1, 2]. Patients in DOSISPHERE-01 were selected based on a dual targeting of tumor and MVI/PVT, crucial to tumor control and resolution of MVI/PVT [1]. Over 40% of patients with MVI/PVT in DOSISPHERE-01 who were treated with multicompartment dosimetry were converted to resection, compared to no patients in the single-compartment dosimetry arm. In the presence of ongoing cirrhotic liver function decline, systemic agents may be considered early on in treatment and in combination with Y-90 following a multidisciplinary review; however, further investigation is needed to better determine specific timing and treatment algorithms [48, 49]. Multicompartment dosimetry is the preferred treatment option in this patient population.

## Future directions

As noted throughout the recommendations, there remain several areas which require additional investigations to better understand optimized patient selection and outcomes. The investigational research areas identified by the committee as high interest and requiring additional data and publications to inform clinical practice are detailed in Table 8. The committee encourages the collection and publication of clinical data to further provide evidence relating to the updated recommendations in these key areas of Y-90 glass microsphere TARE for HCC patients. Here, we briefly discuss two of these areas in which research is currently being or was recently conducted: the use of functional assessments in treatment planning and increasing reproducibility of dosimetric approaches.

**Table 8:** High-priority investigational research areas

	Priority area
1	Evaluation of regional liver function of the FLR using newer imaging modalities to assess pre- and post-Y-90 functional liver volume • Hepatobiliary scintigraphy (^99m^Tc-iminodiacetic acid (HIDA) or ^99m^Tc-mebrofenin) [27] • Eovist/Primovist gadolinium-based contrast agent and MRI imaging
2	Radiation segmentectomy: Evaluation of a streamlined workflow and of clinical outcomes in select populations (due to low lung shunt fraction) Same-day ^99m^Tc-MAA imaging and Y-90 glass microsphere TARE • Elimination of ^99m^Tc-MAA imaging • Use in patients post-hepatectomy • Specific activity impact on rates of complete histopathologic necrosis
3	Radiation lobectomy: • Comparing the outcomes of Y-90 glass microsphere TARE as an alternative to portal vein embolization • Evaluating multicompartment dosimetry
4	Establishing the NTAD limit by • Examining baseline bilirubin impact on a recommended NTAD maximum • Using multicompartment targets in dosimetry planning
5	Studying bilobar disease treatment algorithms addressing: • Sequence of Y-90 glass microsphere TARE, immune checkpoint inhibitors, and/or tyrosine kinase inhibitors • Optimal outcomes of sequential lobar treatment or Y-90 glass microsphere TARE limited to ≤ 70% of the whole liver volume
6	Exploring the sequencing/combination of TARE with systemic therapy
7	Exploring the role of glass-based TARE as first-line treatment in locally advanced HCC (MVI/PVT, no metastases)

### Functional assessments in treatment planning

In a parallel advancement in treatment planning for Y-90 glass microsphere TARE patients with insufficient FLR, functional assessments have been proposed in addition to volume assessments. Traditionally, the timing to undergo resection has focused solely on hypertrophy of the contralateral lobe; however, mounting evidence suggests a role for hepatobiliary scintigraphy to assess regional adequate liver function in confirming treatment candidacy [27–29]. Though the time course of function and volume recovery are parallel, functional recovery lags behind volume. Functional assessment may better drive key clinical decisions regarding treatment success, such as follow-up duration, the need for additional Y-90 treatment, and surgical timing or additional observations. Additional investigation is necessary to confirm the utility of functional assessment in making such treatment decisions.

### Variability and reproducibility of dosimetry

One oft-cited concern with using more complex dosimetry-based approaches is the prediction reproducibility in the treatment phase; however, there are now several tools available to help address these challenges. Several commercial dosimetry software options are available, streamlining calculation of multicompartment dosimetry. Advancements in catheter technique in addition to refinement of the use of ^99m^Tc-MAA imaging to estimate Y-90 glass microsphere distribution have led to improved clinical utility [50–53]. NTAD exhibits better reproducibility, which provides confidence in its use as a safety threshold. Although reproducibility in TAD may be suboptimal, it has been shown to be predictive of patient outcomes (such as tumor response and overall survival) in DOSISPHERE-01, TARGET, and other recent single-center publications [1, 2, 50, 51, 53]. To date, publications evaluated all patients to ascertain reproducibility. However, it may be appropriate to screen out patients in whom multicompartment tumor dosimetry predictions may be unreliable; in those cases, defaulting to MIRD is recommended. Recent publications note that variability may be driven by a limited sample size of published data, operator experience, and variability of tumor flow (and hence T:N) in the presence of multifocal disease, suggesting further optimization of patient selection is needed to improve accuracy and reproducibility of multicompartment dosimetry [52].

## Conclusions

While Y-90 glass microsphere TARE is a key tool in the HCC treatment arsenal, appropriate patient selection, multidisciplinary review, and consideration of alternative or combination treatment in the algorithm are critical to achieving optimal patient outcomes. A number of advancements have been incorporated into the updated HCC treatment recommendations for Y-90 glass microsphere TARE presented here in an effort to improve patient selection, toxicity profiles, and outcomes.

## Data Availability

All data reviewed in the creation of these recommendations can be found in the published literature, per the manuscript’s bibliography.

## Abbreviations

ALBI: Albumin-bilirubin
BCLC: Barcelona Clinic Liver Cancer
CT: Computed tomography
FLR: Future liver remnant
HBS: Hepatobiliary scintigraphy
HCC: Hepatocellular carcinoma
HIDA: Hepatobiliary iminodiacetic acid
MR: Magnetic resonance
MVI/PVT: Macrovascular invasion/portal vein thrombosis
NTAD: Normal tissue–absorbed dose
TAD: Tumor-absorbed dose
TARE: Transarterial radioembolization
Y-90: Yttrium-90
